# Dynamic Distribution of Skin Microorganisms in Donkeys at Different Ages and Various Sites of the Body

**DOI:** 10.3390/ani13091566

**Published:** 2023-05-07

**Authors:** Qingshan Ma, Yunshuang Yue, Xiyan Kou, Wanting Hou, Mingyu Wang, Xihao Yang, Guiqin Liu, Yan Li, Changfa Wang

**Affiliations:** 1 Research Institute of Donkey High-Efficiency Breeding and Ecological Feeding, College of Agronomy, Liaocheng University, Liaocheng 252000, China; horsegreenhill@163.com (Q.M.); kxy19990113@163.com (X.K.); hwt95233@163.com (W.H.); wangmingyu_1222@163.com (M.W.); yxh15166588905@163.com (X.Y.); guiqinliu@lcu.edu.cn (G.L.); 2 College of Food Science and Nutritional Engineering, National Engineering Research Center for Fruit and Vegetable Processing, Key Laboratory of Fruit and Vegetable Processing Ministry of Agriculture, Engineering Research Centre for Fruit and Vegetable Processing, Ministry of Education, China Agricultural University, Beijing 100083, China; daisy_1022@126.com

**Keywords:** skin microbiota, donkeys, dorsal, abdomen, different ages, environment

## Abstract

**Simple Summary:**

Considerable evidence suggests that skin microbiota communities are critical for maintaining health and skin homeostasis. However, to date, studies on the skin microorganisms of donkeys are surprisingly rare. Therefore, the present study investigated the dynamic changes in commensal microbial communities (bacteria and fungal composition) on the skins of healthy donkeys throughout the growing period. Alpha and beta diversity analysis showed that bacterial and fungal communities on the skin display obvious age-related variations, especially at 1 month and 48 months of the growing period. Notably, these changes are also affected by the living environment. Our data could provide insights into microbial involvement in skin disorders and give useful information for improving health management and maintaining the healthy skin of Dezhou donkeys.

**Abstract:**

Considerable evidence suggests that the skin microbiota is not only important and complex in humans and other mammals but also critical for maintaining health and skin homeostasis. To date, studies on the skin microorganisms of donkeys are surprisingly rare. To investigate the dynamic changes in commensal microbial communities on the skins of healthy donkeys throughout the growing period, skin and soil samples were collected from 30 healthy Dezhou donkeys (ranging from 1, 6, 12, 24 to 48 months of age) and their corresponding breeding sheds on the farm. All samples were analysed for high-throughput sequencing of the 16S rRNA and ITS to characterize the skin microbiota of healthy donkeys and compare the differences in skin microbiota among donkeys of different ages. There were notable differences in the proportions of various genera (including bacteria and fungi) between dorsal and abdominal skin with increasing age. The comparison of the skin microbial communities among these groups revealed that *Staphylococcus* was mainly enriched in the early growing stage (1 and 6 months), while the relative abundance of *Streptococcus* was higher in both the 1- and 48-month-old age groups. Moreover, some bacteria and commensal fungi, such as *Staphylococcus* and *Trichosporon*, were found to be positively correlated between the skin and the environment. This is the first study to investigate the dynamic changes in skin microbiota diversity and composition in donkeys of different ages and at different sites of the body. Furthermore, this study provides insights into the dynamic alterations in skin microbes during a donkey’s growth and characterizes the profiles of bacterial and fungal communities across a donkey’s body regions (dorsal and abdomen).

## 1. Introduction

The skin, as the most exposed organ of human and animals, is a complex and dynamic ecosystem that is inhabited by trillions of bacteria, fungi, archaea and viruses. The interaction between the skin microbiota and the host involves not only mutualism but also pathogenicity. Recently, it has been confirmed that the skin microbiota not only coexists with our immune defence network but also modifies immunity, affecting skin health and participating in a variety of skin diseases [1]. *Malassezia* spp., a fungus present on healthy skin, can be stimulated by other factors to exert pathogenic effects and has the ability to cause the development of seborrhoeic dermatitis [2]. Therefore, skin commensal microbial communities have attracted increasing attention in recent years from the perspective of better understanding dermatological diseases and developing new therapies for skin-related diseases.

Donkeys (Equus asinus), a popular species throughout the world, are primarily used as beasts of burden, but occasionally, they also act as a meat source or as pets. Although closely related to horses and able to produce sterile hybrids with them, donkeys are frequently given lower quality care than horses [3]. Therefore, diseases in donkeys are often observed late, especially skin disease. Since most donkeys are neglected, traumatic skin injuries and serious skin diseases are frequent. It has been reported that some of the main skin diseases in donkeys, such as dermatophilosis, staphylococcal infections and ringworm, are caused by the skin microbiota [4,5]. Furthermore, the establishment and colonization of normal microflora is highly driven by the ecology of the skin surface, which is variable depending on endogenous host factors and exogenous environmental factors. Therefore, the donkey’s skin microbiome formed at birth changes gradually with ageing, and any imbalance in the microbiota composition is closely linked to skin diseases [5]. *Staphylococcus*, *Streptococcus pyogenes* and *Trichosporon* are examples of pathogenic microbes that play a role in infectious skin diseases [6,7]. *Staphylococcus epidermidis*, a very common commensal bacterium of the skin, is now regarded as an important opportunistic pathogen [8]. However, previous studies have also reported that *S. epidermidis* may play a probiotic role by preventing colonization of the host by more severe pathogens [9]. Additionally, studies have demonstrated that *S. epidermidis* promotes disease and causes a great number of infections, such as coagulase-negative staphylococci (CoNS), and thetic joint and surgical site infections [10,11]. *Streptococcus equi*, one of most common bacterial pathogens, frequently causes strangle in horses, and is a highly contagious upper respiratory infection in horses. It has been reported that the *S. equi* subspecies pathogen is often resistant to antibiotic treatment making antibiotics ineffective; hence, there is a need for better, improved and optimized techniques for the treatment of *S. equi*-induced infection [12]. The development of molecular approaches for characterizing microbial diversity has dramatically improved our knowledge of the skin microbiome, subsequently drawing attention to the host–microorganism relationship and its relevance to skin disease. However, despite increasing concerns for donkeys’ skin health, such studies on donkeys are still incipient. Additionally, there are few studies regarding how the skin microbiota varies throughout the whole life period of donkeys. Likewise, skin diseases and the relationship between the skin and the commensal microbiota in donkeys are largely unknown. Therefore, it is essential to investigate the changes in commensal microbial communities on the skin of donkeys throughout the growing period.

To identify the changes in donkeys’ skin microbiota throughout the growing period and whether these changes are related to skin diseases, this study evaluated the dynamic alteration of skin commensal microbial communities in donkeys of 1 month to 48 months of age. The results will enhance the understanding of the dynamic variation in the skin microbiota, provide insights into microbial involvement in skin disorders and give useful information for improving health management and maintaining the healthy skins of Dezhou donkeys.

## 2. Materials and Methods

### 2.1. Animals

All animal procedures followed commercial management practice and were reviewed and approved by Animal Welfare Committee of Liaocheng University (Permit No. DFG21010103-1). A total of 30 healthy Dezhou donkeys ranging from 1, 6, 12, 24 and 48 months of age were selected and divided into five groups: the 1-month age group, 6-month age group, 12-month age group, 24-month age group and 48-month age group. Each group consisted of six donkeys. All donkeys were raised under normal conditions at a Dezhou donkey original breeding farm with corresponding (age-related) feeding management. One-month-old donkeys were fed with fresh donkey milk. Donkeys aged 6 months were weaned and fed with a commercial concentrate feed (Hekangyuan Group Co., Ltd., Jinan, China). The remaining donkeys were all fed with the same commercial concentrate feed. All donkeys were provided feed twice daily (08:00 am and 16:00 pm) at 0.25 and 1.5% of their body weight, respectively, and the ratio of wheat straw to concentrate was 60:40 in the diet. All the donkeys were allowed to drink water *ad libitum* throughout the breeding period. No antibiotics were used in donkeys for at least 3 months before sampling.

### 2.2. Sample Collection

**Figure 1:**
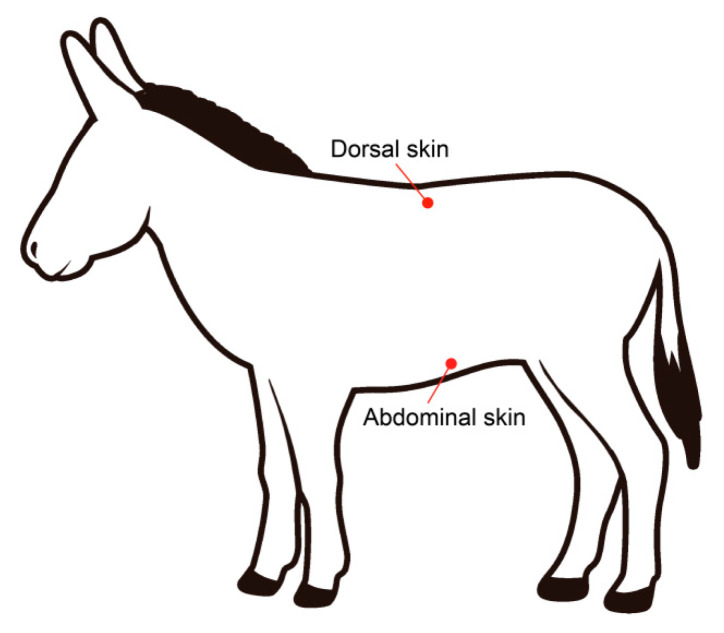
Body sites of the microbiota isolated from Dezhou donkey skins.

One sample was taken from each donkey skin: dorsal skin and abdominal skin (Figure 1). To collect the depth bacteria and fungi on the skin surface, the hair on the skin surface of the donkeys was cut off. Sterile cotton swabs were dipped in sterile saline to moisten the swab before collection. Then the cotton swab was moved back and forth across the length of a square approximately 10 × 10 cm^2^, horizontally and vertically for 15 s, respectively, three swabs used in the same pattern for each collection. Each sample was stored in sterilized 2 mL tubes, and then snap frozen in liquid nitrogen. All frozen samples were stored with dry ice and submitted to the laboratory of Majorbio Bio-Pharm Technology Co., Ltd. for further analysis.

Soil samples were collected from five breeding sheds housing the different aged donkeys on the farm. Soil was randomly sampled from 3 points in each breeding shed. For each point, about 100–150 g soil (0–5 cm depth from the surface) was collected using plastic spoons and then placed in a sterile bag. Plastic spoons were disinfected with 75% alcohol. All the sterile bags containing soil were immediately stored at −80 °C for further analysis.

### 2.3. DNA Extraction and PCR Amplification

Genomic DNA was extracted from skin and breeding environment samples using the E.Z.N.A.^®^ soil DNA Kit (Omega Bio-tek, Norcross, GA, USA), and V3-V4 region of the bacterial 16S and ITS1 region of fungal ITS rRNA gene were amplified, respectively. The 16S rRNA gene was amplified using an ABI GeneAmp^®^ 9700 PCR thermocycler (ABI, Foster City, CA, USA), with primer pairs 338F (5′-ACTCCTACGGGAGGCAGCAG-3′) and 806R(5′-GGACTACHVGGGTWTCTAAT-3′). A reaction containing 4 μL of 5 ×TransStart FastPfu buffer, 2 μL of 2.5 mM dNTPs, 0.8 μL of forward and reverse primer (5 μM), 0.4 μL of TransStart FastPfu DNA Polymerase and 10 ng template DNA in 20 μL total volume was used. Additionally, the ITS rRNA gene was amplified by an ABI GeneAmp^®^ 9700 PCR thermocycler, using the primers ITS1F (5′- CTTGGTCATTTAGAGGAAGTAA-3′) and ITS2R (5′-GCTGCGTTCTTCATCGATGC-3′). A PCR reaction contains 10 μL of 2 × Pro Taq, 0.8 μL of forward and reverse primer (5 μM), and 10 ng template DNA in 20 μL total volume. The PCR reactions were carried out and the products were extracted from 2% agarose gel and purified using the AxyPrep DNA Gel Extraction Kit (Axygen Biosciences, Union City, CA, USA). The purified amplicons were pooled in equimolar and paired-end sequences on an Illumina MiSeq PE300 platform (Illumina, San Diego, USA) at Majorbio company (Shanghai, China). The raw sequence data were transmitted to the NCBI Sequence Read Archive (SRA) database, and the accession numbers were PRJNA940337 and PRJNA940505 for bacterium and fungi, respectively.

### 2.4. Analysis of 16S and ITS Amplicon Sequencing Data

Fastq data were demultiplexed and quality filtered using fastp and merged by FLASH. All data that did not meet the criteria were identified and discarded. The data were further denoised and clustered into operational taxonomic units (OTUs) with 97% similarity cutoff using UPARSE version 7.1. After identifying and removing the chimeric sequences, the taxonomy of each OTU representative sequence was assigned by the RDP Classifier against Silva 16S rRNA database using a confidence threshold of 70%. To minimize the bias due to variation in sequencing depths, data were rarefied to 22,340 and 31,666 sequences for 16S rRNA and ITS of each sample, respectively. Alpha and beta diversities were calculated by QIIME version 1.9.1 software. Alpha diversity was calculated using the abundance-based coverage estimator (ACE), Shannon diversity index, Simpson index and bias-corrected Chao estimator (Chao 1), referring to richness and uniformity of the skin microbiota within the samples. Beta diversity was evaluated by calculating the Bray–Curtis distances between samples using QIIME software and visualized by principal component analysis (PCA). Linear discriminant analysis (LDA) coupled with effect size (LEfse) was used to identify the most significantly differential taxa among different groups. The threshold of LDA scores was set to 4 for 16S and ITS sequences. Spearman correlation tests were used to evaluate the correlation between the skin microbiota and the breeding environment microbiota.

### 2.5. Statistical Analysis

The data were analyzed on the Majorbio Cloud platform (www.majorbio.com, accessed on 21 August 2022). Wilcoxon rank-sum test was used to estimate the variance in alpha diversity of skin microbiota. Based on the Bray–Curtis distance, we used PCA plots to visualize the differences in bacterial community composition. Correlation analysis of the top most abundant taxa in skin and environment was performed using Spearman’s correlation coefficients in R (version 3.3.1). A *p*-value of < 0.05 was considered to be significantly correlated.

## 3. Results

### 3.1. Skin Microbiome Structure of Donkeys Aged 1 Month to 4 Years

**Figure 2:**
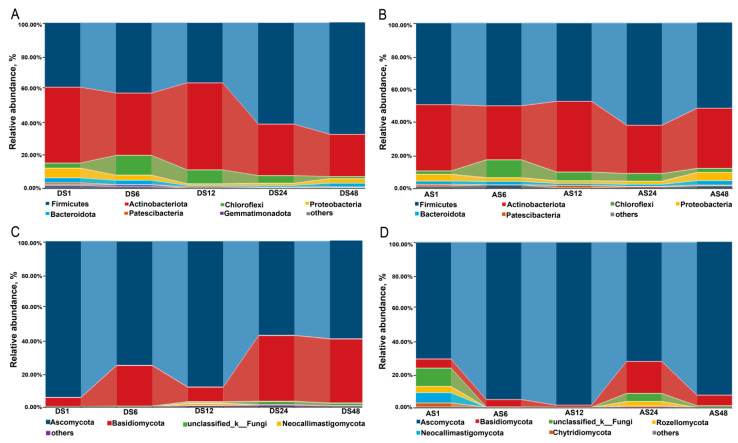
Comparative analyses of the taxonomic composition of the microbial community in the skin of Dezhou donkeys. Each component of the cumulative bar chart indicates a phylum and the alteration trends between adjacent bars for the current revealed taxa are indicated by the interval belts. The relative abundances of the bacteria at the phylum level in dorsal skin (**A**) and abdominal skin (**B**) from different ages; dorsal (**C**) and abdomen (**D**) skin fungi composition at the phylum level. (DS1, sampled from 1 month dorsal skins; DS6, sampled from 6 month dorsal skins; DS12, sampled from 12 month dorsal skins; DS24, sampled from 24 month dorsal skins; DS48, sampled from 48 month dorsal skins; AS1, sampled from 1 month abdomen skins; AS6, sampled from 6 month abdomen skins; AS12, sampled from 12 month abdomen skins; AS24, sampled from 24 month abdomen skins; AS48, sampled from 48 month abdomen skins).

Skin and breeding environment microbiome analysis was conducted using 16S and ITS rRNA sequencing of 1-, 6-, 12-, 24- and 48-month-old Dezhou donkeys. A total of 3,819,208 and 5,252,453 features and 1,591,892,407 and 1,141,495,519 reads were obtained from 75 samples with mean lengths of 416 and 217 for 16S rRNA and ITS sequencing, respectively. The composition of the microbial communities varied significantly between dorsal and abdominal skin (*p* < 0.05; Figure 2 and Figure S1). At the phylum level, the dorsal and abdominal skin were both dominated by Firmicutes, Actinobacteriota, Chloroflexi, Proteobacteria and Bacteroidota (Figure 2A,B). At the genus level, the dorsal skin was dominated by *Planococcus*, *Corynebacterium* and *Salinicoccus*, and the abdominal skin was dominated by *Staphylococcus*, *Corynebacterium* and *Salinicoccus* (Figure S1A,B). There were notable differences in the proportions of various genera between dorsal and abdominal skin with increasing age (Figure S1A,B).

The fungal composition results showed that the dominant phyla in the dorsal skin were Ascomycota and Basidiomycota, which accounted for more than 95% of the relative abundance (Figure 2C). Furthermore, the relative abundance of Basidiomycota increased in the donkeys from 1 month to 24 months of age and tended to reach a steady-state level at 48 months of age, with the relative abundance of *Ascomycota* decreasing from 1 month to 24 months of age and tending to reach a steady-state level at 48 months of age (Figure 2C). The dominant phyla in the abdominal skin were Ascomycota and Basidiomycota in donkeys of 1 month to 48 months of age. Moreover, the relative abundance of Ascomycota increased within the first 12 months and peaked at 12 months of age. In contrast, the abundance of Basidiomycota dropped in the donkeys from 6 months to 12 months of age and rebounded at 24 months (Figure 2D). In addition, the levels of dominant genera in the dorsal skin, including *Wallemia*, *Alternaria* and *unclassified_f_Saccharomycetales_fam_Incertae_sedis*, varied among donkeys of different ages (Figure S1C). The abdominal skin was dominated by *Sarocladium*, *unclassified_f__Microascaceae* and *Issatchenkia* (Figure S1D). There were significant differences in the proportions of various genera across body regions and ages (Figure S1C,D).

### 3.2. Age-Related Differences in Alpha Diversity

Significant differences were found in the number of Chao-estimated OTUs among the five age groups (Table 1). The observed Chao 1 and ACE of dorsal skin bacterial indices were highest in the 48-month-old group. Conversely, the above two indices were significantly decreased from the 1-month to the 24-month-old age groups. The Shannon index significantly decreased with age, and it was highest in the 1-month-old age group. However, it was significantly higher in the 48-month-old age group than in the 24-month-old age group, thus indicating an age-related modification in the distribution of bacterial communities (Table 1). Additionally, the Simpson index in the 1-month-old age group was lowest, which was consistent with the results for the Shannon index. Detailed information is provided in Table 1.

The Chao 1 and ACE indices of dorsal skin fungi were lowest in the 1-month-old age group, while the 24-month-old age group had the highest values. However, no significant difference was observed among the 6-month, 12-month and 24-month-old age groups. The Shannon index in the 1-month-old age group was lower than that in the other groups, and it tended to become stable at 6 months, thus indicating age-related stability in the distribution of the fungal communities. Moreover, the Simpson index in the 1-month-old age group was highest, which was consistent with the results for the Shannon index (Table 1).

The alpha diversity of the abdominal skin bacteria indicated that the 48-month-old age group showed a significantly higher number of Chao-estimated OTUs than the other groups, revealing no difference in the richness within samples among the four age groups except for in the 48-month-old age group (Table 1). Consistently, the Shannon index was highest in the 48-month-old age group, suggesting an age-related modification in the distribution of bacterial communities (Table 1). Moreover, the Simpson index in the 1-month-old age group was highest, which was consistent with the results for the Shannon index. Significant differences were found in the Shannon index for abdominal skin fungi, revealing differences in the diversity within samples among the five age groups. In addition, the Simpson index in the 1- and 24-month-old groups was lower than that in the other three groups, which was consistent with the results for the Shannon index (Table 1).

### 3.3. Age-Related Differences in Beta Diversity

**Figure 3:**
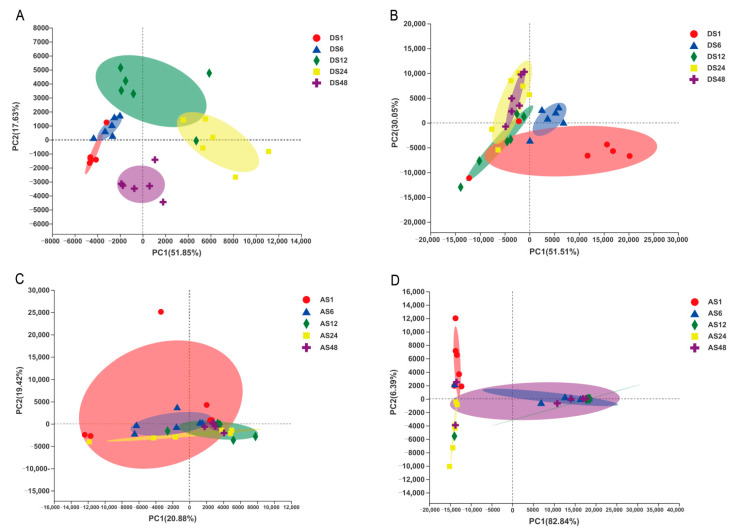
The principal components analysis (PCA) of samples from different ages, dorsal and abdominal skins. Bacterial samples (**A**) and fungal samples (**B**) from dorsal skins; bacterial samples (**C**) and fungal samples (**D**) from abdominal skins. (DS1, sampled from 1 month dorsal skins; DS6, sampled from 6 month dorsal skins; DS12, sampled from 12 month dorsal skins; DS24, sampled from 24 month dorsal skins; DS48, sampled from 48 month dorsal skins; AS1, sampled from 1 month abdomen skins; AS6, sampled from 6 month abdomen skins; AS12, sampled from 12 month abdomen skins; AS24, sampled from 24 month abdomen skins; AS48, sampled from 48 month abdomen skins).

The beta diversity results showed significant differences in the skin bacterial structure in different groups. The dorsal skin bacteria were notably segregated as donkey age increased, especially among the 1-, 12- and 48-month-old groups, but were less dispersed between the 1- and 6-month-old and 12- and 24-month-old groups (Figure 3A). In contrast, obvious segregation of dorsal skin fungi was found among the 1-month-old, 6-month-old and older age groups, but less segregation was found in the 12-month-old, 24-month-old and 48-month-old groups (Figure 3B). However, data from the abdominal skin showed that the bacteria were clustered together (Figure 3C). Additionally, we found that the fungal community exhibited a similar tendency in that there was low dispersal across all donkey age groups (Figure 3D).

### 3.4. Microbial Community Composition Differences in Donkey Skin with Age

**Figure 4:**
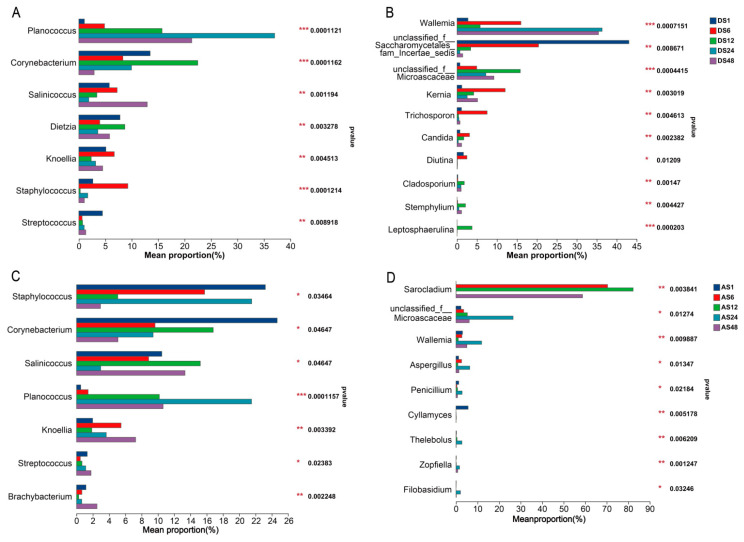
The different bacterial and fungal composition of the skin microbiota in Dezhou donkeys at different ages. The relative abundance of the genera *Planococcus*, *Corynebacterium*, *Salinicoccus*, *Dietzia*, *Knoellia*, *Staphylococcus* and *Streptocossus* in dorsal skins (**A**); the relative abundance of the genera *Wallemia*, *Unclassified_f_saccharomycetales_fam_incertae_sedis*, *Unclassified_f_Microascaceae*, *Kernia*, *Trichosporon*, *Candida*, *Diutina*, *Cladosporium*, *Stemphylium* and *Leptosphaerulina* in dorsal skins (**B**); the relative abundance of the genera *Staphylococcus*, *Corynebacterium*, *Salinicoccus*, *Planococcus*, *Knoellia*, *Streptocossus* and *Brachybacterium* in abdominal skins (**C**); the relative abundance of the genera *Sarocladium*, *Unclassified_f_Microascaceae*, *Wallemia*, *Aspergillus*, *Cyllamyces*, *Thelebolus*, *Zopfiella* and *Filobasidium* in abdominal skins (**D**). (DS1, sampled from 1 month dorsal skins; DS6, sampled from 6 month dorsal skins; DS12, sampled from 12 month dorsal skins; DS24, sampled from 24 month dorsal skins; DS48, sampled from 48 month dorsal skins; AS1, sampled from 1 month abdomen skins; AS6, sampled from 6 month abdomen skins; AS12, sampled from 12 month abdomen skins; AS24, sampled from 24 month abdomen skins; AS48, sampled from 48 month abdomen skins. Kruskal–Wallis H test, *p* * < 0.05, *p* ** < 0.01, *p* *** < 0.001; *n* = 6).

The relative abundance of the skin microbes on the dorsal skins at the phylum and genus levels is shown in Figure S2A and Figure 4A. Additionally, we found that the dominant bacterial phyla in the dorsal skins were Firmicutes and Actinobacteriota, which accounted for more than 80% of the relative abundance. After comparing the differences among all groups, it was shown that the relative abundance of Firmicutes increased notably as donkey age increased, and it was highest in the 48-month-old age group (*p* < 0.05). However, the relative abundance of Actinobacteriota was markedly decreased from the 1-month-old to the 48-month-old age groups (*p* < 0.05). We also found that at the phylum level, Chloroflexi tended to increase obviously before 6 months of age but declined slightly thereafter (*p* < 0.05). The relative abundances of both Proteobacteria and Bacteroidota tended to decline from 1 month to 12 months of age but increased slightly thereafter (*p* < 0.05, Figure S2A).

At the genus level, the dorsal skin was dominated by *Planococcus*, *Corynebacterium* and *Salinicoccus* (Figure 4A). As age increased, the relative abundance of *Planococcus* tended to augment before 24 months of age, and it was highest in the 24-month-old age group (*p* < 0.05). Both the relative abundances of *Corynebacterium* and *Dietzia* were higher in the 12-month-old age group than in the other groups (*p* < 0.05).

The dominant dorsal skin fungal phyla were Ascomycota and Basidiomycota, which accounted for more than 95% of the relative abundance (Figure 2B). Furthermore, the differences in dorsal fungal phylotypes showed that the relative abundance of Ascomycota was higher in the 1- and 12-month-old age groups than in the other groups, while the relative abundance of Basidiomycota was higher in the 24-month-old and 48-month-old age groups than in the other groups (*p* < 0.05). The relative abundances of *Kernia*, *Trichosporon* and *Candida* were higher in the 6-month-old age group than in the other groups. The 12-month-old age group had the highest *Cladosporium*, *Stemphylium* and *Leptosphaerulina* abundances (*p* < 0.05, Figure 4B).

We found that the abdominal skin was also dominated by Firmicutes and Actinobacteriota, which accounted for more than 90% of the relative abundance. Furthermore, the analysis of differences in abdomen microbiota indicated that the relative abundance of Chloroflexi tended to increase obviously before 6 months of age but declined slightly thereafter (*p* < 0.05, Figure S2C), which was consistent with the trend in dorsal skin. The relative abundance of Proteobacteria was highest in the 48-month-old age group, and the 6-month-old age group had the highest Gemmatimonadota and Acidobacteriota (*p* < 0.05). In addition, the relative abundance of Patescibateria was highest in the 12-month-old age group (*p* < 0.05). At the genus level, the abdominal skin was dominated by *Staphylococcus*, *Corynebacterium* and *Salinicoccus* (Figure 4C). Interestingly, the relative abundances of *Staphylococcus* and *Corynebacterium* were highest in the 1-month-old age group. Additionally, both the 1-month-old and 48-month-old age groups had more *Streptococcus* than the other groups. The results for abdomen fungi indicated that the dominant phyla were consistent with those of dorsal skin, which accounted for more than 75% of the relative abundance (Figure S2D). In addition, the dominant abdominal skin genera, including *Sarocladium*, *unclassified_f__Microascaceae* and *Issatchenkia*, varied among donkeys of different ages. The differences in abdomen fungal phylotypes showed that the 1-month-old age group had more phyla, including the phyla Rozellomycota, Neocallimastigomycota and Zoopagomycota (Figure S2D). Interestingly, the 24-month-old age group had the most genera, including the genera *unclassified_f_Microascaceae*, g-*Wallemia*, g-*Aspergillu*, etc. (Figure 4D).

### 3.5. Taxonomic and Functional Changes in Skin Microbiomes among the Five Age Groups

**Figure 5:**
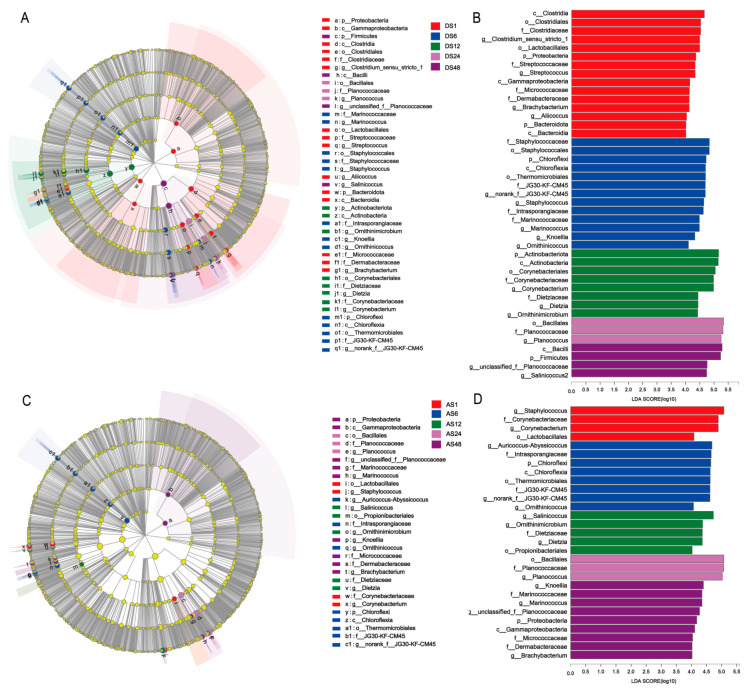
LEfSe analysis of the skin microbial composition of Dezhou donkeys at different ages. Cladogram obtained from the LEfSe method, indicating the phylogenetic distribution of the dorsal skin microbiota (**A**) and abdomen skin microbiota (**C**); histogram of the LDA scores, showing the most differentially abundant taxa of dorsal skin (**B**) and abdomen skin (**D**) among different age groups (LDA score >4.0, *n* = 6). (DS1, sampled from 1 month dorsal skins; DS6, sampled from 6 month dorsal skins; DS12, sampled from 12 month dorsal skins; DS24, sampled from 24 month dorsal skins; DS48, sampled from 48 month dorsal skins; AS1, sampled from 1 month abdomen skins; AS6, sampled from 6 month abdomen skins; AS12, sampled from 12 month abdomen skins; AS24, sampled from 24 month abdomen skins; AS48, sampled from 48 month abdomen skins).

The histogram of the LDA scores revealed a pronounced differential abundance among the five groups. Regarding the dorsal skin, the results showed that the relative abundances of the phyla Proteobacteria and Bacteroidota, orders Clostridiales and Lactobacillales, families Clostridiaceae, Streptococcaceae and Micrococcaceae, and genera *Clostridium_sensu_stricto_1*, *Streptococcus* and *Brachybacterium* were highly enriched in the 1 month-old group; the phylum Chloroflexi, order Staphylococcales and Thermomicrobiales, family Staphylococcaceae, and genus *Staphylococcus* were highly enriched in the 6-month-old group; the phylum Actinobacteriota, order Corynebacteriales, families Corynebacteriaceae and Dietziaceae, and genera *Corynebacterium*, and *Dietzia* were highly enriched in the 12-month-old group; the order Bacillales, family Planococcaceae, and genus *Planococcus* were highly enriched in the 24-month-old group; and the phylum Firmicutes and the genera *unclassified_f__Planococcaceae* and *Salinicoccus* were highly enriched in the 48-month-old group (Figure 5A,B).

Regarding the abdominal skin, the data showed that *Staphylococcus* and *Corynebacterium* were greatly enriched in the 1-month-old group; *Auricoccus-Abyssicoccus*, *norank_f__JG30-KF-CM45* and *Ornithinicoccus* were enriched in the 6-month-old group; *Salinicoccus* and *Ornithinimicrobium* were enriched in the 12-month-old group; *Planococcus* was enriched in the 24-month-old group; and *Knoellia*, *Marinococcus* and *unclassified_f__Planococcaceae* were enriched in the 48-month-old group (Figure 5C,D).

The fungal taxonomy revealed the contribution of each taxon to the previously identified differences among the five age groups (Figure S3). Regarding the dorsal skin, a high contribution of the genera *unclassified_f__Saccharomycetales_fam_Incertae_sedis* was found in the 1-month-old age group. In addition, six other genera (*Kernia*, *Trichosporon*, *Aspergillus*, *Candida*, *unclassified_f__Dipodascaceae* and *Diutina)* were identified, contributing to the differences observed in the 6-month-old age group. In the 12-month-old age group, the relative abundance of the genera *unclassified_f__Microascaceae*, *Leptosphaerulina* and *Clonostachys* contributed to the difference from other age groups. The genera *Wallemia* and *unclassified_c__Sordariomycetes* contributed to the age-related difference observed for the 24-month-old and 48-month-old groups, respectively (Figure S3A,B). Consistent with the dorsal skin, the results of the abdominal skin in the 1-month-old group showed that *unclassified_f__Saccharomycetales_fam_Incertae_sedis* and *Cyllamyces* contributed to the differences from other groups. A high contribution of the genus *Sarocladium* was found in the 6-month-old age group. Additionally, eight other genera (*unclassified_f__Microascaceae*, *Wallemia*, *Aspergillus*, *unclassified_c__Sordariomycetes*, *Thelebolus*, *Thermomyces*, *Penicillium* and *Fusicolla*) were identified, contributing to the differences observed in the 24-month-old age group. In the 48-month age group, the relative abundance of the genus *Purpureocillium* contributed to the pronounced difference from other groups (Figure S3C,D).

### 3.6. Bacterial and Fungal Functional Characteristics

**Figure 6:**
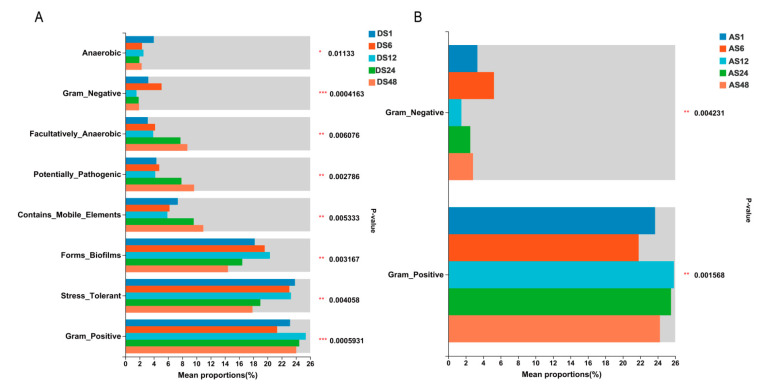
The distinct functional composition of the identified bacteria in Dezhou donkeys during different growth stages. The abundance of Anaerobic, Gram_negative, Facultatively_anaerobic, Potentially_pathogenic, Contains_mobile_elements, Forms_biofilms, Stress_tolerant and Gram_positive in dorsal skin (**A**); the abundance of Gram_negative and Gram_positive in abdomen skins (**B**). (DS1, sampled from 1 month dorsal skins; DS6, sampled from 6 month dorsal skins; DS12, sampled from 12 month dorsal skins; DS24, sampled from 24 month dorsal skins; DS48, sampled from 48 month dorsal skins; AS1, sampled from 1 month abdomen skins; AS6, sampled from 6 month abdomen skins; AS12, sampled from 12 month abdomen skins; AS24, sampled from 24 month abdomen skins; AS48, sampled from 48 month abdomen skins. Kruskal–Wallis H test, *p* * < 0.05, *p* ** < 0.01, *p* *** < 0.001; *n* = 6).

Additionally, we predicted the functional composition of the identified bacteria using BugBase. The abundances of microbes with the potentially pathogenic phenotype and the facultative anaerobic phenotype gradually increased (Figure 6). The proportion of microbes with the stress-tolerant phenotype decreased in the 6-month-old group; the proportion of microbes that could form biofilms initially increased in the 6-month-old group and then decreased in the 24-month-old group, while the proportion of Gram-positive microorganisms decreased first in the 6-month-old group and then started to increase in the 12-month-old group (Figure 6A). Moreover, the abundance of microbes with the anaerobic phenotype was highest in the 1-month-old group, while the proportion of Gram-negative microorganisms was initially increased in the 6-month-old group, followed by a decrease in the 12-month-old group. The abundance of microbes with the mobile element phenotype decreased first and then increased at 24 months of age (Figure 6A). Similarly, the functional composition of the abdominal skin bacteria showed that the abundance of microbes with the Gram-negative phenotype in the 6-month-old group was higher, while the abundance of microbes with the Gram-positive phenotype was lower than in the other groups. In the 12-month-old group, the proportion of microbes with the Gram-negative phenotype was lowest, while the proportion of Gram-positive microbes was higher than in other groups (Figure 6B).

**Figure 7:**
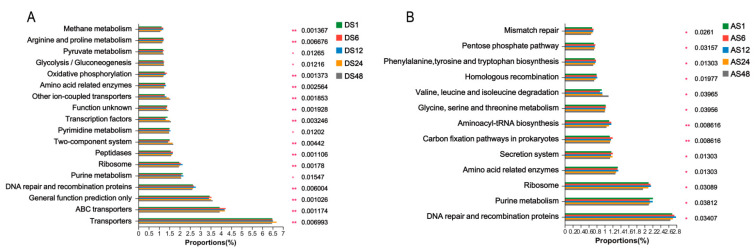
Significantly different distribution of level 3 of predicted functional categories among groups. Eighteen items of functional category among groups in dorsal skin (**A**); thirteen items of functional category among groups in abdomen skin (**B**). (DS1, sampled from 1 month dorsal skins; DS6, sampled from 6 month dorsal skins; DS12, sampled from 12 month dorsal skins; DS24, sampled from 24 month dorsal skins; DS48, sampled from 48 month dorsal skins; AS1, sampled from 1 month abdomen skins; AS6, sampled from 6 month abdomen skins; AS12, sampled from 12 month abdomen skins; AS24, sampled from 24 month abdomen skins; AS48, sampled from 48 month abdomen skins. *p* * < 0.05, *p* ** < 0.01; *n* = 6).

Furthermore, PICRUSt was used to evaluate the metabolic function of the microbial communities based on the gene sequence at Kyoto Encyclopedia of Genes and Genomes (KEGG) taxonomy level 3. Generally, the potential functions of the dorsal skin bacteria of donkeys were mainly in transporters, DNA repair and recombination proteins, purine metabolism, ribosomes and peptidases. The older groups had lower activities of methane metabolism, arginine and proline metabolism, oxidative phosphorylation, amino-acid-related enzymes, peptidases, ribosome and DNA repair and recombination proteins than the younger groups. However, the capacities of other iron-coupled transporters, transcription factors and two-component systems were increased in the older groups (Figure 7A). Similar to the function of dorsal skin bacteria, abdominal skin bacteria were mainly involved in DNA repair and recombination proteins, purine metabolism, ribosome and amino-acid-related enzymes. The older groups had lower activities of mismatch repair, pentose phosphate pathway, amino-acid-related enzymes and DNA repair and recombination proteins than the younger groups, while the capacity of valine, leucine and isoleucine degradation was promoted in the older groups (Figure 7B).

**Figure 8:**
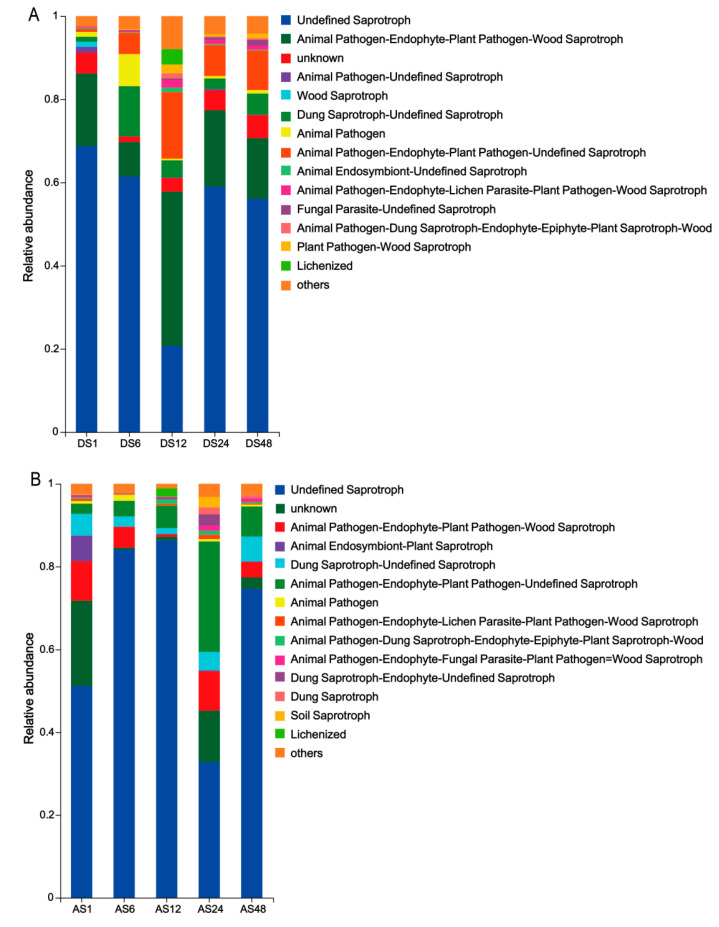
Relative abundance of trophic and guild modes assigned by FUNGuild for fungal communities. Analysis in dorsal skins (**A**) and abdomen skins (**B**). (DS1, sampled from 1 month dorsal skins; DS6, sampled from 6 month dorsal skins; DS12, sampled from 12 month dorsal skins; DS24, sampled from 24 month dorsal skins; DS48, sampled from 48 month dorsal skins; AS1, sampled from 1 month abdomen skins; AS6, sampled from 6 month abdomen skins; AS12, sampled from 12 month abdomen skins; AS24, sampled from 24 month abdomen skins; AS48, sampled from 48 month abdomen skins.

FUNGuild analysis was conducted to determine the predicted functions of the donkey skin fungal communities. We found that saprotrophs were the dominant fungal taxa observed in the dorsal skin (Figure 8A). The relative abundance of undefined saprotrophs in the 12-month-old group was lowest, but the animal pathogen saprotrophs and lichenized were highest. However, the results for the abdominal skin were in contrast with those obtained from the dorsal skin (Figure 8B).

### 3.7. Correlations between the Skin Microbiota and Breeding Environment Microbiota

The interaction between the skin commensal microbiota and environmental microbiota was investigated. At the genus level, several microbes were significantly altered on the dorsal skin and in the environment. Interestingly, there was a significantly positive correlation between *Staphylococcus* levels in the skin and those in the environment (Figure S4A). Additionally, fungi such as *Penicillium*, *Trichosporon* and *Diutina* were positively correlated between the dorsal skin and the environment (Figure S4B). Furthermore, there were positive correlations in the levels of *Trichosporon* and *Candida* between the abdominal skin and the environment (Figure S4D).

## 4. Discussion

It has been reported that the composition of the skin microbiome can change as age increases in humans [13]. However, related information on these alterations has not been studied in donkeys. Therefore, herein, we investigated the differences in skin microbiota composition in Dezhou donkeys of different ages. The present study indicates dramatic dynamic changes in skin microbiota diversity and composition in donkeys of different ages and at different sites of the body, especially at 1 and 48 months of age. Our results provide insights relevant to donkey feeding management and suggest that changes in age and living environment can influence the skin microbiome, skin health and disease in donkeys.

In the present study, we found striking influences on the diversity and taxonomic composition of the skin microbiota of Dezhou donkeys at different points of the growing period. The results revealed higher alpha diversity on the dorsal skin of the 1- and 48-month-old groups than in other groups, with a significant age-related change in the distribution and richness of the dorsal skin microbiota. In contrast, the alpha diversity data of the dorsal fungi indicated that the richness of the microbiota was lowest in the 1-month-old group and the diversity showed an age-related stable trend from 6 months, which was consistent with previous studies showing lower bacterial and fungal diversity in younger humans [14,15]. Similarly, the results obtained for the alpha diversity examination on abdominal skins reflected a low diversity in 1-month-old donkeys and a high diversity in 48-month-old donkeys, as with the trends for dorsal skin richness. This result is slightly different from that obtained from the dorsal skin and may be due to the natural characteristics of equine rolling, which causes differences in exposure to ground dust, facilitating different interactions with the environment between the two sites on the body.

In addition, beta diversity examination demonstrated significant differences in the bacterial and fungal composition between young (1 and 6 months) and adult donkeys (12, 24 and 48 months), while the 12-, 24- and 48-month-old groups mostly clustered together. This result reflected that the composition of the skin microbiota was variable during the early stage of growth and then gradually became similar and stabilized as growth occurred. The dynamic age-related variation in α and β diversity revealed that growth is an important factor in shaping the skin microbiota.

In addition to microbial diversity, bacterial and fungal species with significantly different abundances among the five age groups were identified in the skin. In the present study, Firmicutes and Actinobacteriota were the dominant phyla in the donkey skin microbiota in all groups. Furthermore, we observed that the relative abundance of the phylum Firmicutes was highest in the 48-month-old age group, whereas the abundance of Actinobacteriota was lowest in the 48-month-old age group. *Planococcus* spp. and *Corynebacterium* spp. were enriched in the dorsal skin of the young donkeys compared with the adult donkeys, which could be correlated with the place of residence, skin condition and diseases associated with growing stage [16], consistent with the findings of previous studies [17,18]. An increasing number of studies have verified the direct interactions between the host immune system and the skin microbiota [19,20]. *Staphylococcus epidermidis*, a ubiquitous human skin colonizer, was generally recognized as an opportunistic pathogen in the previous literature; however, recent studies have indicated that *S. epidermidis* could interact with other microorganisms to modulate immunological signals and stimulate innate host defence or directly interfere with the overgrowth of pathogenic microorganisms to exert a protective role in mucosal immune defence [21,22]. Our findings showed that *Staphylococcus* spp. was highly abundant in the abdominal skin of the 1- and 48-month-old age groups, which suggests that except for the potential probiotic roles of *S. epidermidis* in promoting homeostasis of the skins, more attention should be given to the possible infection caused by these potentially pathogenic genera in young and adult donkeys. *Streptococcus* spp., acting as an opportunistic pathogen and contributing to strangles disease in horses [12], was also abundant in donkey abdominal skin of the 1- and 48-month-old age groups in this study, which is consistent with previous reports on donkey oral cavities [23]. Therefore, the presence of these bacteria with high abundance in the 1- and 48-month-old age groups may suggest that donkeys at these two stages are more susceptible to the influence of disease, which underscores the need to pay special attention to donkeys during these periods.

At the phylum level, Ascomycota and Basidiomycota were the predominant fungi on donkey dorsal skin, which was consistent with the findings of a previous study on the donkey caecum and colon, which had the highest abundance of Ascomycota and Basidiomycota [24]. At the genus level, *Wallemia*, *Alternaria*, *unclassified_f_Saccharomycetales_fam_Incertae_sedis*, *Issatchenkia*, *Sarocladium* and *unclassified_f__Microascaceae* were the predominant genera on dorsal and abdominal skin in the current study. The genus *Wallemia* is a group of xerophilic filamentous fungi with the ability to secrete a series of glycosidases that are able to degrade both α- and β-linked glycosidic bonds among di- and polysaccharides [25,26]. *Alternaria* spp., filamentous Ascomycetes, are often present on plants as pathogens and endophytes and in soil as saprophytes [27]. Furthermore, *unclassified_f_Saccharomycetales_fam_Incertae_sedis* and *Issatchenkia* were reported to have the potential ability to produce organic acid [28]. In addition, sequencing evidence revealed that *Sarocladium* caused human clinical cases, including mycetomas, serious diseases affecting the lungs and catheter-related bloodstream infections [29,30]. The fungal community composition results also showed differences among different age groups. The abundance of *Kernia*, *Trichosporon* and *Candida* was greatest in the 6-month-old group. In contrast, the *unclassified_f__Microascaceae*, *Cladosporium*, *Wallemia*, *Aspergillus*, *Penicillium*, *Thelebolus* and *Filobasidium* in 12- and 24-month-old donkey skin were greater than in other groups. Moreover, it has been reported that these genera mostly have the ability to produce various highly active enzymes, including esterase, amylase and protease, which may enable skin microorganisms to promote the good health of donkeys [31,32]. Nevertheless, as these fungi have scarcely been found in herbivores, their functional significance and metabolism in donkey skin ecosystems require further investigation and confirmation. This study revealed that the bacterial and fungal communities on the skin displayed obvious age-related variations, which is consistent with findings from a previous study in humans [15].

Evidence indicates that specific microbes on the skin are associated with skin health and diseases, and are even linked with several systemic diseases [33]. We further identified specific functional pathways exerted by the microbial community in donkeys at different ageing stages and varied sites on the body using functional prediction analysis. Additionally, our data showed dynamic changes in microbial functional redundancy at different ages and various sites on the body in healthy donkeys. For instance, the younger skin samples had higher biofilm formation and oxidative stress tolerance than the adult skin samples, especially at the dorsal site, which indicated that certain resident microorganisms can withstand some environmental stressors, such as oxidative stress, in the young stage. Consistent with this finding, the skin-residing microbiota in a high-altitude group were found to have higher oxygen tolerance than those in a low-altitude group, and could adapt well to the extreme environment and colonization in the host by forming a biofilm at a high altitude [34]. It has been reported that biofilm formation by the skin-residing indigenous microbiota may play a key role in the prevention of skin infection [35,36] and may help maintain skin health. However, biofilm formation is also considered a global regulator of pathogenic bacterial virulence factors [37], which also indicates that special attention should be given to pathogenic bacteria, especially in animals of younger stages (immature skin status). Interestingly, the abundances of microbes with potentially pathogenic phenotypes gradually increased with age. Moreover, the results from predicting metabolic functions revealed that some functional orthologues involved in DNA repair and energy supplementation (e.g., oxidative phosphorylation) as well as in collagen synthesis (arginine and proline metabolism) were decreased in dorsal samples of adult donkeys. In addition to age and site factors, the environment is considered to play an important role in affecting the skin microbiota [38]. Notably, we observed that the abundance of *Staphylococcus* in skin was positively correlated with the environment (especially in the dorsal site), suggesting that strict management of the living environment for donkeys is needed. Furthermore, some skin commensal fungi (such as *Trichosporon*) were positively correlated between the skin and the environment. However, the FUNGuild tool showed that the composition of fungal functional guilds among the five groups mostly involved pathogenicity-related functions, indicating that fungal functions need to be studied further. To more deeply understand the functional capacity of the skin microbial community, metagenomic studies on these populations will be needed.

## 5. Conclusions

In conclusion, bacterial and fungal communities on the skin displayed obvious age-related variations, especially at 1 month and 48 months of the growing period. Moreover, the present study showed that some bacteria and commensal fungi, such as *Staphylococcus* and *Trichosporon* were positively correlated between the skin and the environment, indicating that the skin microbiota is a very dynamic entity influenced by the external environment. Together, these findings suggested that the structure and function of the skin microbiota of donkeys are variable throughout the early stage of growth and then gradually tend to be similar and stabilize with increasing age. Our results provide new insights relevant to feeding management concerning host-associated microbiome diversity and stability and provide guidance in understanding the variations in donkey skin microbiota in health and disease at different stages of growth. Although we found that the diversity and composition of many bacteria and fungi varied significantly over the growing period in donkeys, their functional significance and metabolism in donkey skin ecosystems require further investigation and confirmation.

## Figures and Tables

**Table 1:** Alpha diversity indices of different groups.

Index		DS					AS				
		1 Month	6 Months	12 Months	24 Months	48 Months	1 Month	6 Months	12 Months	24 Months	48 Months
Bacteria	sobs	1285.17 ± 29.80 ^a^	1042.17 ± 63.59 ^b^	802.83 ± 68.20 ^c^	757.83 ± 61.29 ^c^	1069.17 ± 65.18 ^b^	982.67 ± 140.40 ^b^	921.33 ± 89.30 ^b^	884.17 ± 63.26 ^b^	847.67 ± 47.71 ^b^	1287.33 ± 82.02 ^a^
	ACE	1896.90 ± 153.95 ^ab^	1963.50 ± 97.40 ^a^	1586.37 ± 116.74 ^b^	1573.74 ± 104.50 ^b^	2204.33 ± 86.12 ^a^	1680.27 ± 179.90 ^b^	1730.79 ± 108.02 ^b^	1754.32 ± 84.43 ^b^	1652.48 ± 75.89 ^b^	2087.35 ± 69.63 ^a^
	Chao	1734.37 ± 90.11 ^a^	1588.41 ± 76.60 ^a^	1284.37 ± 88.29 ^b^	1223.46 ± 89.23 ^b^	1781.74 ± 76.04 ^a^	1416.23 ± 138.63 ^b^	1423.90 ± 98.71 ^b^	1415.06 ± 93.76 ^b^	1368.06 ± 64.78 ^b^	1884.82 ± 64.79 ^a^
	Shannon	4.75 ± 0.06 ^a^	4.32 ± 0.19 ^ab^	3.84 ± 0.19 ^c^	3.39 ± 0.17 ^d^	4.05 ± 0.11 ^bc^	3.23 ± 0.48 ^b^	3.76 ± 0.34 ^ab^	3.71 ± 0.35 ^ab^	3.49 ± 0.17 ^ab^	4.40 ± 0.10 ^a^
	Simpson	0.03 ± 0.00 ^c^	0.04 ± 0.01 ^bc^	0.07 ± 0.02 ^b^	0.13 ± 0.02 ^a^	0.05 ± 0.01 ^bc^	0.24 ± 0.07 ^a^	0.08 ± 0.02 ^b^	0.11 ± 0.05 ^ab^	0.12 ± 0.03 ^ab^	0.04 ± 0.00 ^b^
Fungus	sobs	135.17 ± 35.90 ^c^	337.33 ± 14.14 ^ab^	325.50 ± 21.48 ^ab^	369.33 ± 43.43 ^a^	277.83 ± 10.94 ^b^	199.33 ± 58.08 ^a^	197.83 ± 29.86 ^a^	90.17 ± 17.62 ^b^	200.17 ± 17.71 ^a^	174.17 ± 12.02 ^ab^
	ACE	171.53 ± 48.16 ^c^	424.95 ± 14.91 ^ab^	376.29 ± 29.97 ^ab^	448.60 ± 58.15 ^a^	318.76 ± 15.30 ^b^	233.73 ± 68.43	250.60 ± 35.00	192.71 ± 19.46	221.37 ± 15.05	208.11 ± 11.98
	Chao	171.10 ± 50.13 ^c^	417.70 ± 16.45 ^ab^	377.50 ± 29.59 ^ab^	448.69 ± 57.52 ^a^	319.05 ± 15.05 ^b^	230.40 ± 68.58	249.74 ± 34.58	136.98 ± 19.78	225.36 ± 17.84	206.71 ± 10.53
	Shannon	1.80 ± 0.31 ^b^	3.19 ± 0.08 ^a^	2.76 ± 0.34 ^a^	2.86 ± 0.24 ^a^	3.02 ± 0.12 ^a^	2.95 ± 0.37 ^a^	1.34 ± 0.46 ^b^	0.63 ± 0.51 ^b^	3.21 ± 0.18 ^a^	1.63 ± 0.58 ^b^
	Simpson	0.39 ± 0.08 ^a^	0.09 ± 0.01 ^b^	0.23 ± 0.08 ^b^	0.17 ± 0.03 ^b^	0.13 ± 0.02 ^b^	0.16 ± 0.05 ^b^	0.62 ± 0.13 ^a^	0.83 ± 0.14 ^a^	0.13 ± 0.03 ^b^	0.55 ± 0.16 ^a^

AS, abdomen skin; DS, dorsal skin; sobs, observed species richness; ACE, abundance-based coverage estimator. Data on the alpha diversity indices were subjected to one-way ANOVA followed by Duncan multiple comparison using the SPSS statistical software package (version 22). The variability in the results is expressed as the mean ± standard error. ^a, b, c, d^ Means with different letters were considered significantly different at *p* < 0.05.

## Data Availability

These sequence data have been submitted to the GenBank databases under accession number PRJNA940337 and PRJNA940505.

## Supplementary Materials

The following supporting information can be downloaded at: https://www.mdpi.com/article/10.3390/ani13091566/s1, Figure S1 Comparative analyses of the taxonomic composition of the microbial community in the skin of Dezhou donkeys. Each component of the cumulative bar chart indicates a genus and the alteration trends between adjacent bars for the current revealed taxa are indicated by the interval belts. The relative abundances of the bacteria at the genus level in dorsal skin (A) and abdomen skin (B) from different ages; dorsal skin (C) and abdomen skin (D) fungi composition at the genus level. (DS1, sampled from 1 month dorsal skins; DS6, sampled from 6 month dorsal skins; DS12, sampled from 12 month dorsal skins; DS24, sampled from 24 month dorsal skins; DS48, sampled from 48 month dorsal skins; AS1, sampled from 1 month abdomen skins; AS6, sampled from 6 month abdomen skins; AS12, sampled from 12 month abdomen skins; AS24, sampled from 24 month abdomen skins; AS48, sampled from 48 month abdomen skins). Figure S2 The different bacterial and fungal compositions of the skin microbiota in Dezhou donkeys at different ages. The relative abundance of the phyla Firmicutes, Actinobacteriota, Chloroflexi, Proteobacteria, Bacteroidota, Patescibacteria and Myxococcota in dorsal skin (A); the relative abundance of the phyla Ascomycota, Basidiomycota, Neocallimastigomycota, Rozellomycota, Zoopagomycota, Aphelidiomycota and Monoblepharomycota in dorsal skin (B); the relative abundance of the phyla Chloroflexi, Proteobacteria, Patescibacteria, Deinococcota, Gemmatimonadota, Acidobacteriota and Myxococcota in abdominal skin (C); the relative abundance of the phyla Ascomycota, Basidiomycota, Rozellomycota, Neocallimastigomycota, Zoopagomycota, Aphelidiomycota and Kickxellomycota in abdominal skin (D). (DS1, sampled from 1 month dorsal skin; DS6, sampled from 6 month dorsal skin; DS12, sampled from 12 month dorsal skin; DS24, sampled from 24 month dorsal skin; DS48, sampled from 48 month dorsal skin; AS1, sampled from 1 month abdomen skin; AS6, sampled from 6 month abdomen skin; AS12, sampled from 12 month abdomen skin; AS24, sampled from 24 month abdomen skin; AS48, sampled from 48 month abdomen skin. Kruskal–Wallis H test, *p ** < 0.05, *p *** < 0.01, *p **** < 0.001; *n* = 6). Figure S3 LEfSe analysis of the skin microbial composition of Dezhou donkeys at different ages. Cladogram obtained from the LEfSe method, indicating the phylogenetic distribution of the dorsal skin fungi (A) and abdomen skin fungi (C); histogram of the LDA scores, showing the most differentially abundant taxa of dorsal skin (B) and abdomen skin (D) among different age groups (LDA score > 4.0, *n* = 6). DS1, sampled from 1 month dorsal skin; DS6, sampled from 6 month dorsal skin; DS12, sampled from 12 month dorsal skin; DS24, sampled from 24 month dorsal skin; DS48, sampled from 48 month dorsal skin; AS1, sampled from 1 month abdomen skin; AS6, sampled from 6 month abdomen skin; AS12, sampled from 12 month abdomen skin; AS24, sampled from 24 month abdomen skin; AS48, sampled from 48 month abdomen skin. Figure S4 Correlation plot showing Spearman’s correlations between the skin microbiota and the breeding environment microbiota (core genera top 20). Correlation analysis of dorsal skin bacteria (A) and fungi (B); correlation analysis of abdomen skin bacteria (C) and (D) fungi. * *p* < 0.05, ** *p* < 0.01, *** *p* < 0.001.
