# Supplementary figures and images for: Dynamic Distribution of Skin Microorganisms in Donkeys at Different Ages and Various Sites of the Body
Source: Animals (Basel). 2023 May 7;13(9):1566. doi: 10.3390/ani13091566 (PMC10177048; doi:10.3390/ani13091566)

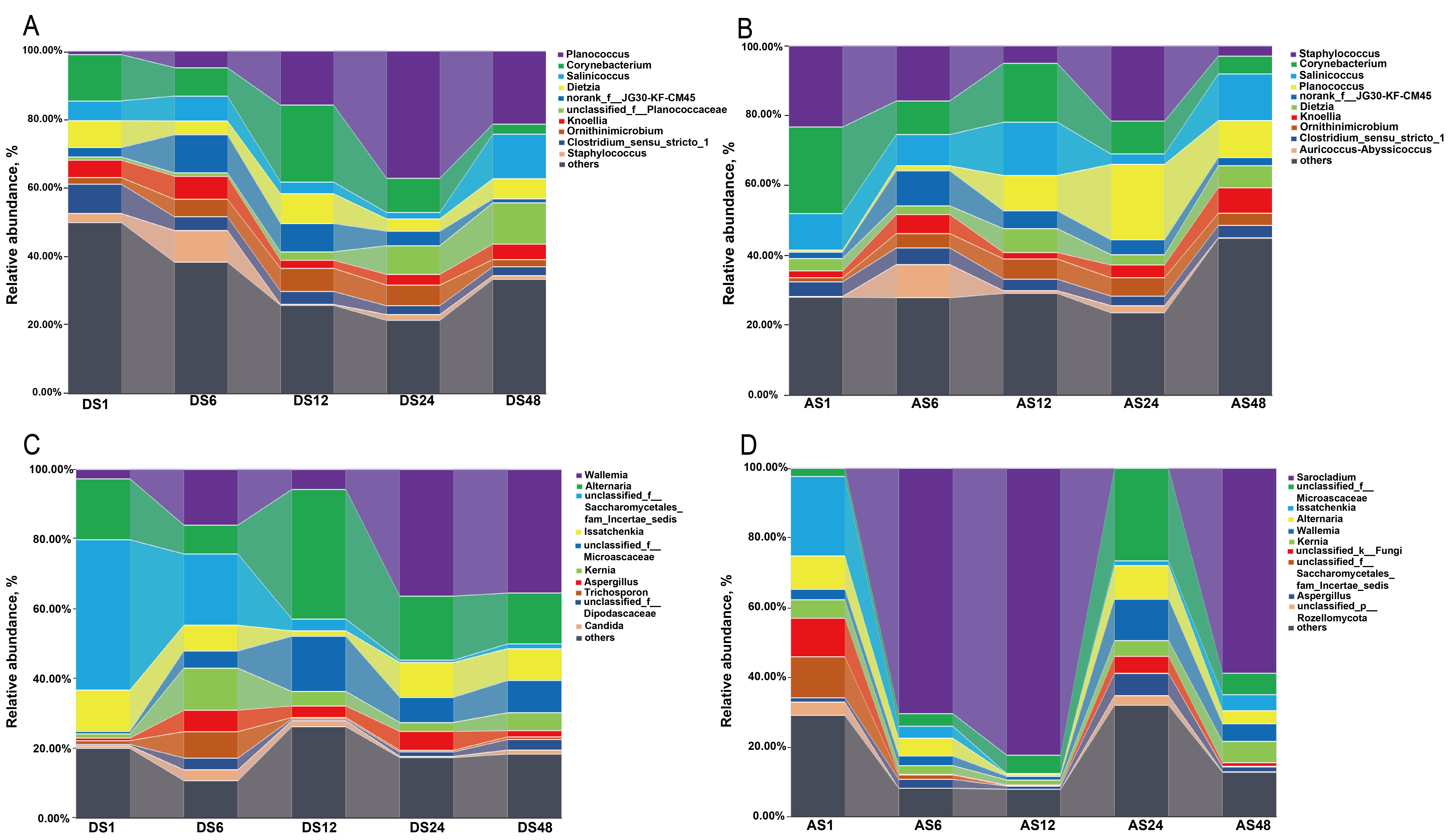

Supplement: Supplementary file 1 [file animals-13-01566-s001.zip › Figure S1.tif]

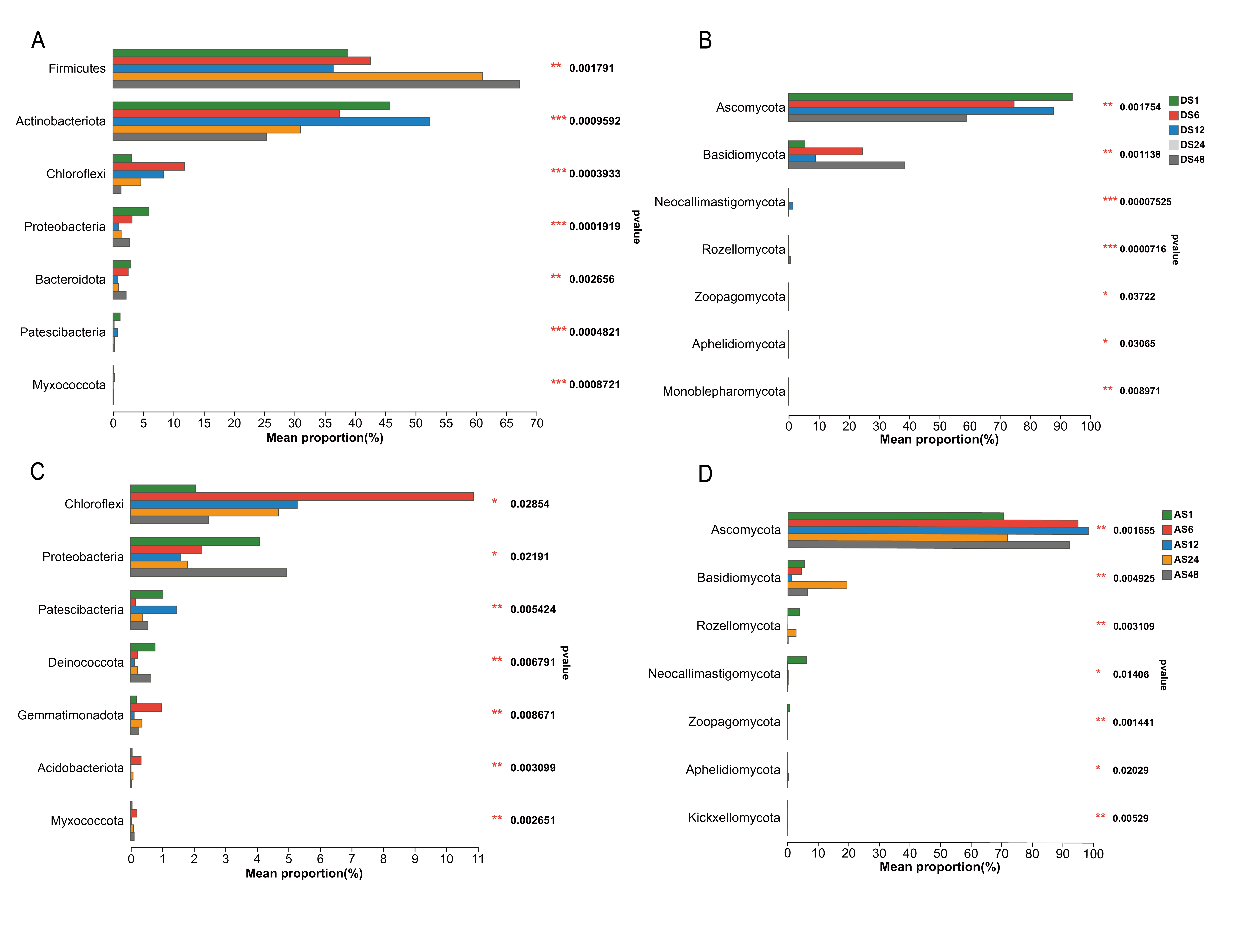

Supplement: Supplementary file 1 [file animals-13-01566-s001.zip › Figure S2.tif]

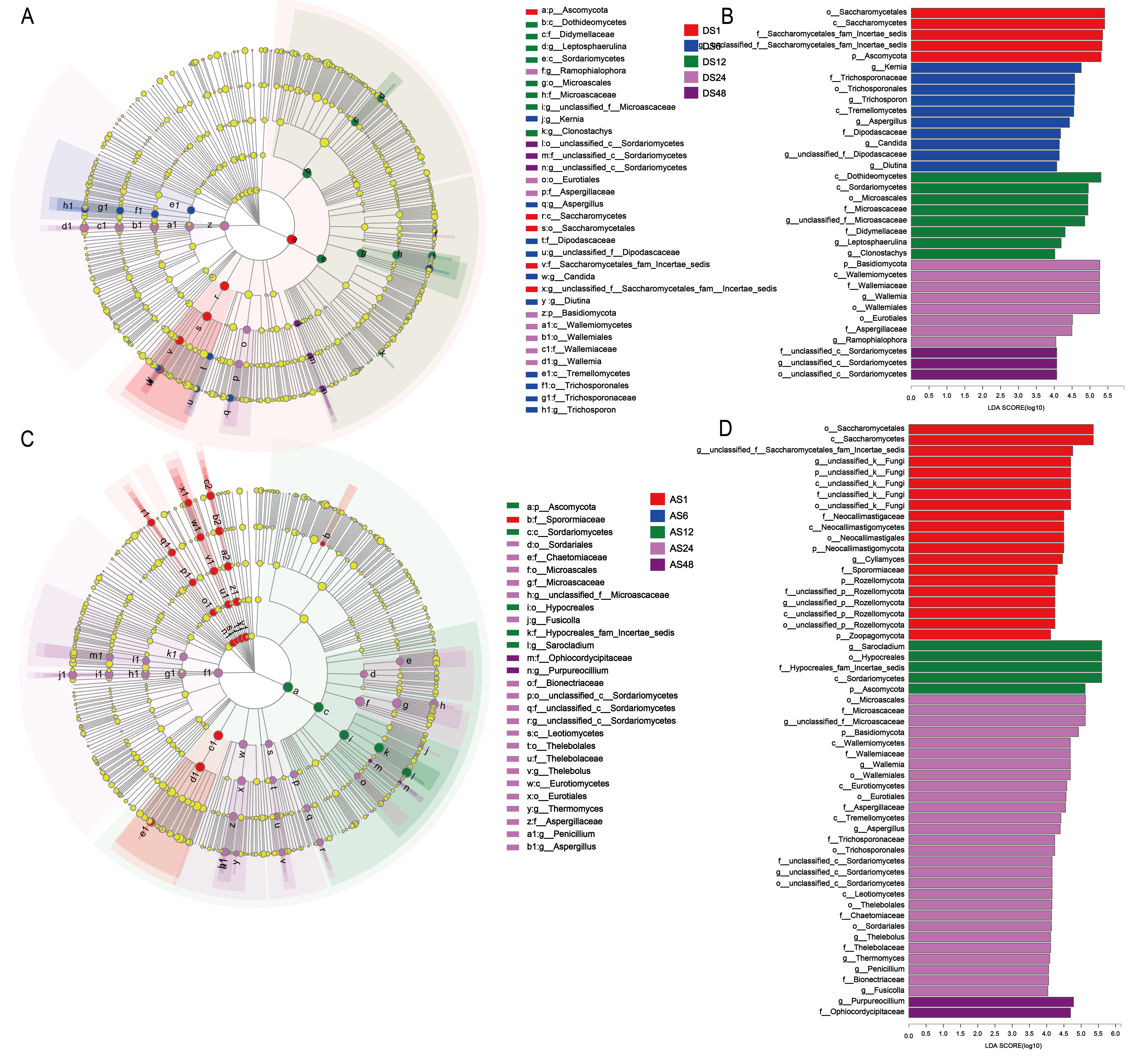

Supplement: Supplementary file 1 [file animals-13-01566-s001.zip › Figure S3.tif]

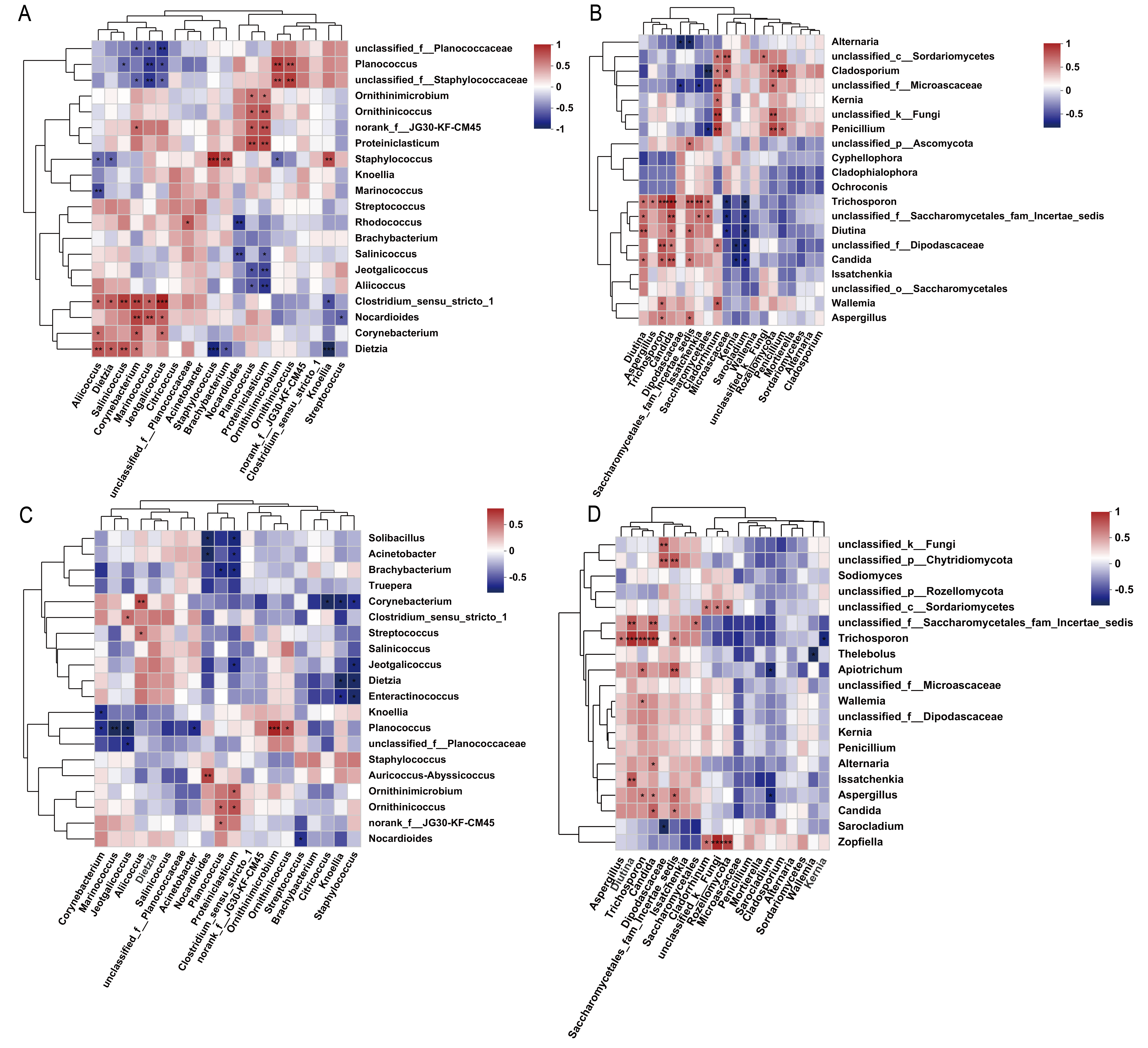

Supplement: Supplementary file 1 [file animals-13-01566-s001.zip › Figure S4.tif]
